# Proof of Efficacy Study to Evaluate an Ayurvedic Formulation in the Treatment of Allergic Rhinitis: An Open Label Randomized Controlled Clinical Trial

**DOI:** 10.7759/cureus.46663

**Published:** 2023-10-08

**Authors:** Vaidya B Prakash, Yashwant K Rao, Shikha Prakash, Sneha T Sati, Ankita Mohapatra, Neha Negi

**Affiliations:** 1 Ayurveda, Vaidya Chandra Prakash Cancer (VCPC) Research Foundation, Rudrapur, IND; 2 Pediatrics, Ganesh Shankar Vidyarthi Memorial (GSVM) Medical College, Kanpur, IND; 3 Medicine, Padaav - A Specialty Ayurvedic Treatment Centre, Rudrapur, IND; 4 Clinical Research, Vaidya Chandra Prakash Cancer (VCPC) Research Foundation, Rudrapur, IND; 5 Clinical Research, Ganesh Shankar Vidyarthi Memorial (GSVM) Medical College, Kanpur, IND; 6 Clinical Research, Padaav - A Specialty Ayurvedic Treatment Centre, Rudrapur, IND

**Keywords:** tnss, montelukast, levocetirizine, immbo, ige, ayurveda, allergic rhinitis

## Abstract

Background: Allergic rhinitis is largely treated by using antihistamines and nasal sprays, either alone or in combination. However, these measures ease out the symptoms but do not address causative factors, and have their share of side effects and limitations. An Ayurvedic herbo-mineral formulation, IMMBO, has been reported to be effective in treating allergic rhinitis.

Objective: The present study was carried out to evaluate the efficacy, safety, and tolerability of the Ayurvedic herbo-mineral formulation in comparison with a fixed-dose combination of levocetirizine and montelukast.

Method: This was a randomized, comparative, clinical study carried out on 250 patients at a medical college in India. The patients were enrolled according to the eligibility criteria of the study and randomized into two groups, to receive either Ayurvedic herbo-mineral formulation, IMMBO, or a combination of levocetirizine and montelukast for 28 days. Total nasal symptom score (TNSS) and Immunoglobulin E (IgE) were calculated for evaluation of efficacy parameters.

Result: At the end of therapy both IMMBO and levocetirizine and montelukast combination showed significant improvement in TNSS in both treated population and per protocol population. The IMMBO group had a statistically higher reduction in TNSSs compared to the levocetirizine + montelukast group (-5.70 vs. -3.31; p<0.01). There was a statistically significant difference in the reduction of IgE levels between the groups (-351.54 vs. -208.79; p<0.05).

Conclusion: The findings of this study establish prima facie evidence about the efficacy and safety of Ayurvedic formulation. However, the said Ayurvedic formulation needs to be further developed scientifically.

## Introduction

Rhinitis is characterized by inflammation of the mucus membrane of the nose marked especially by rhinorrhea, nasal congestion, itching, and sneezing [1]. Rhinitis has broadly been categorized as allergic, infectious, non-allergic, and non-infectious rhinitis [2]. Allergic rhinitis is further categorized into seasonal (intermittent) and perennial (persistent) allergic rhinitis [3]. Seasonal allergic rhinitis (hay fever) has been briefly described in the Islamic text of the 9th century. During the 15th century various terminologies were used for rhinitis such as Catarrh coined from the work Katarrhen which means flow down. Subsequently, European texts described allergic rhinitis in the 16th century. The 19th century witnessed the wide emergence of hay fever with the industrialization of Westernized countries where it was a common condition and now both types of rhinitis are global diseases [4].

Ayurveda is an ancient medical system in India that describes many aspects of health; prevention and treatment of diseases. *Charak Samhita *has been considered the oldest treatise of Ayurveda, originating from 2000 BC, and written in the Sanskritlanguage [5]. Charakhas mentioned the disease called *Pratishyay*, marked by the main symptoms of sneezing and a watery nose. Later on, *Sushrut Samhita* in the 6th century, *Ashtang Hridaya* in the 7-8th century,* Madhav Nidan* in the 7-8th century, *Ashtang Sangraha* in the 9-10th century, *Chakradatt *in 11th century, *Bhav Prakash* in 16th century and *Bhaishajya Ratnawali *in 18th century has described *Pratishyay *as an independent disorder [6-11]. Ayurvedic literature has emphasized treating *Pratishyay *by eliminating the causative factors and adopting appropriate lifestyle and medicine otherwise it converts into *Dusht Pratishaya *[12]. Its symptoms are similar to those of persistent allergic rhinitis/sinusitis/chronic obstructive pulmonary disease. 

Allergic rhinitis is managed by avoidance of allergies, maintaining hygienic conditions, and consuming antihistamines in conventional medicine [13]. Corticosteroids are also used mostly in nasal sprays to bring instant relief [14]. As per modern medicines, combining levocetirizine with montelukast treats allergies effectively. Levocetirizine is an antiallergen that blocks histamine, relieving runny nose, watery eyes, and sneezing, and montelukast obstructs leukotrienes, reducing inflammation in the airways and nose, further alleviating allergy symptoms [15].

Ayurveda is widely practiced in India alongside Western medicine and other alternative systems of medicine. Registered Ayurvedic practitioners can use and formulate Ayurvedic remedies in their clinical practice without needing a drug manufacturing license [16]. In 1997, a North India-based Ayurvedic clinic discovered the therapeutic effect of a herbo-mineral formulation (HMF) in treating persistent allergic rhinitis. HMF effectively relieves acute and persistent allergic rhinitis in various Indian Ayurvedic clinics. IMMBO is a judicious combination of eighteen herbs (*Cedrus deodara, Curcuma longa, Cypus rotundus, Emblica officinalis, Emblica ribes, Holarrhena antidysentrica, Picrorrhiza kurroa, Berberis aristata, Piper longum, Piper longum (Root), Piper nigrum, Plumbago zeylanica, Saussurea lappa, Terminallia belerica, Terminallia chebula, Zingiber officinalis, Boerhavia diffusa, Operculina terpathum*)and *Mandoor Bhasma*. *Mandoor* is an iron ore that gets converted into a complex mineral form with mainly fayalite and hematite. 

This study was conducted to evaluate the efficacy and tolerability of a novel therapeutic intervention IMMBO in comparison with a conventional combination of levocetirizine and montelukast in patients diagnosed with allergic rhinitis as per standard Allergic Rhinitis and its Impact on Asthma (ARIA) 2019 diagnostic criteria [17].

## Materials and methods

Study design

This was a prospective, randomized, active-controlled, comparative, parallel-group clinical study conducted in a tertiary care teaching hospital (GSVM Medical College, Kanpur) in India. The study was planned for 250 patients with allergic rhinitis. Patients satisfying the eligibility criteria were enrolled and randomized in a 1:1 ratio to receive IMMBO (60 mg/kg/day) or a fixed-dose combination of levocetirizine 2.5 mg + montelukast 4 mg for a treatment period of 28 days. 

Ethics

The study was conducted in accordance with the ethical principles of the Declaration of Helsinki. It was approved by the Institutional and Ethical Clearance Board of GSVM Medical College, Kanpur, India (Approval no./ID: EC/BMHR/2022/20). The study was also registered with the Clinical Trial Registry of India (CTRI registration No. REF/2022/06/055573). All patients provided their written informed consent prior to participation in the study. 

Patient eligibility criteria

Male and female patients aged between 4 and 60 years who were satisfying the ARIA 2019 diagnostic criteria were enrolled in the study. To be eligible for the study, the patient had to have a history of allergic rhinitis and active symptoms of the disease during enrollment. Patients were required to have any of the following two symptoms of allergic rhinitis, that is sneezing, itchy nose and/or palate, nasal congestion, rhinorrhea, conjunctival hyperemia, and eye-watering and should have had a total nasal symptom score (TNSS) of ≥ 2. TNSS is the sum of scores for nasal congestion, sneezing, nasal itching, and rhinorrhea at each time point. It is calculated using a four-point scale ranging from 0-3 (0 = no symptoms, 1 = mild symptoms that can be easily tolerated, 2 = awareness of symptoms that are bothersome but manageable, and 3 = severe symptoms that are difficult to tolerate and interfere with daily activities). TNSS is calculated by adding the score for each of the symptoms, where a maximum score can be 12. Patients were also required to have serum Immunoglobin E (IgE) levels>100 IU/mL.

Patients with a history of acute or chronic sinusitis within 30 days of the screening visit were excluded from the study. Individuals with a history of rhinitis medicamentosa, non-allergic rhinitis, major structural nasal blockage, nasal polyps, or any other clinically significant nasal anomaly were also excluded from the study. Patients with upper respiratory tract infections including cold and systemic infections within 3 weeks or history of eye surgery or intranasal surgery within 3 months or with severe asthma requiring emergency room treatment within 1 month or hospitalization within 3 months of baseline visit were also excluded. Patients having clinically significant impaired hepatic and renal functions were excluded from the study. Patients with abnormal ECG (conduction delay, abnormal QTc interval) were also excluded. Patients with a history of gastrointestinal, cardiovascular, respiratory, hematological, endocrine, or neurological disorders as well as those receiving immunotherapy within the previous six months were excluded. Patients with allergies to any of the medicines or any of the ingredients of the formulation were excluded from the study. Patients already receiving medications for allergic rhinitis/ conjunctivitis such as antihistaminic, inhaled, oral, parenteral, nasal, and ophthalmic corticosteroids, cromolyn sodium, nedocromil or inhaled anticholinergics, long-acting inhaled β-agonists, Theophylline, and Leukotriene modifiers, decongestants, and anti-inflammatory drugs were excluded from the study. 

Study interventions

Eligible patients of allergic rhinitis were randomized to receive either HMF IMMBO oral powder at a dose of 60 mg/kg/day in three divided doses or a fixed-dose combination of levocetirizine 2.5 mg and montelukast 4 mg once daily for 28 days. Oral suspension of levocetirizine + montelukast was preferred in children of 4 to 11 years of age. Patients above 12 years received tablets of levocetirizine and montelukast combination. The subjects were randomized to study treatments in a 1:1 ratio. During the treatment, patients visited the clinic on day 14 and day 28 (end of the study visit). 

Study procedure

During the study, patients underwent screening at baseline, day 14, and end-of-therapy visits (day 28). During the screening visit patient’s demography, physical examination, vital signs, past medical history, family history of allergic rhinitis, current signs and symptoms of the disease, details of concurrent medications, and lab investigations were recorded to evaluate the eligibility of the patients as per the inclusion-exclusion criteria. Blood samples were also collected during the baseline visit to estimate IgE levels. 

TNSS and IgE levels were captured for eligible patients during baseline visits and patients were randomized to either of the two treatments. On day 14, a follow-up visit for safety assessment and drug dispensing was performed. On day 28 visit, TNSS and IgE levels were performed to asses efficacy, and laboratory investigations were performed for safety assessment. 

Study endpoints

The study endpoints included analyzing and comparing the changes from baseline in TNSS and IgE levels between the IMMBO group and the levocetirizine + montelukast group over the 4-week treatment period.

Safety assessment was based on adverse events and changes in laboratory parameters. An adverse event was defined as any untoward medical occurrence including an abnormal laboratory finding occurring in trial subjects that may or may not be related to the study treatment. Patients were encouraged to report adverse events spontaneously or respond to a general non-directed questionnaire.

Statistics

A sample size of 90 patients in each group was estimated to give more than 85% power to detect a difference of 1.5 in mean change from baseline to Week 4 in TNSS between IMMBO and levocetirizine + montelukast group with a significance level of 0.05 and standard deviation (SD) of 3.3. After allowing 20% dropouts, 112 patients were required in each treatment group. Statistical analysis was performed on all randomized patients who completed 4 weeks of treatment without any protocol violation. Descriptive statistics were used to compare the demographic and baseline disease characteristics. Data was presented in terms of mean ± SD, median, percentiles, or range for continuous variables and percentage for categorical variables. All the patients were compared at baseline for homogeneity using a two-sample t-test or Kruskal-Walli’s test for continuous variables and the Chi-square test or Fisher’s exact test for categorical variables. 

The primary efficacy parameter was the mean change in TNSS at Week 4 from baseline and the difference between the two groups was assessed using a two-sample t-test and within treatment group comparisons were assessed using paired t-test. The secondary efficacy parameter was the mean change in IgE levels at Week 4 from baseline and the difference between the two groups was assessed using a two-sample t-test and within treatment group comparisons were assessed using a paired t-test. Change in ARIA symptoms from baseline to Week 4 was assessed using the Chi-square test for between-group comparison and within-treatment group comparisons were assessed using McNemar’s test. 

Safety parameters were the incidence of adverse events and changes in laboratory parameters from the baseline. A comparison of the incidence of adverse events was to be done using the Chi-square test or Fisher’s exact test. Changes in laboratory parameters from baseline to week 4 were assessed using a two-sample t-test and within treatment group comparisons were assessed using a paired t-test. The level of significance was set at 0.05. Statistical analysis was performed using SAS 9.4 (SAS Institute Inc., Cary, NC).

## Results

This study screened 250 patients with allergic rhinitis from July 22 to December 22. Of these, 224 subjects who met the inclusion-exclusion criteria were enrolled in the study. Twelve subjects withdrew consent prior to enrolment, 11 subjects did not satisfy the eligibility criteria, and 3 subjects were lost to follow-up between screening visit and baseline visit.

**Figure 1:**
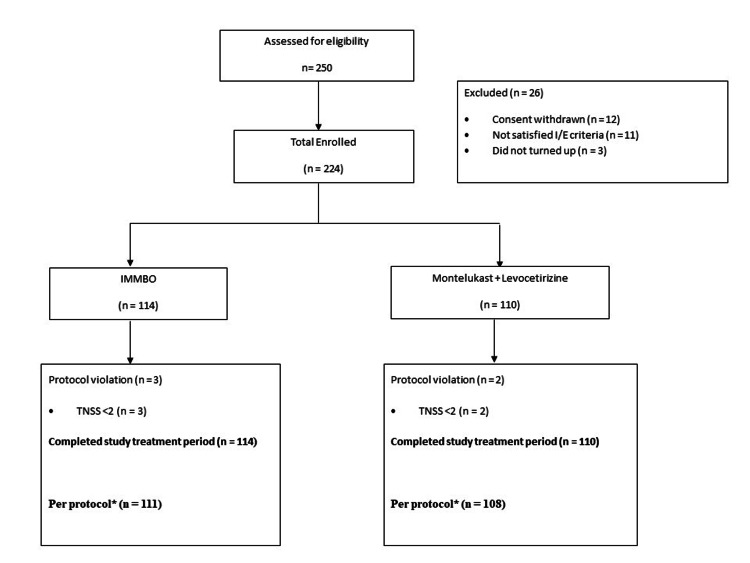
Disposition of study participants *Indicates per protocol population which comprised all enrolled subjects who completed the study without any protocol violations; TNSS: total nasal symptom score; IMMBO: Ayurvedic herbo-mineral formulation

Of these 224 subjects, 114 were randomized to receive IMMBO, and 110 received montelukast + levocetirizine fixed-dose combination. All the subjects in both groups completed the 28-day treatment period. There were 3 protocol violations in the IMMBO group and 2 protocol violations in the montelukast + levocetirizine group (Figure 1). All 224 patients who completed the 28-day treatment period were included in the efficacy and safety analysis. Efficacy analysis was also performed on a per protocol population of 219 patients (that excludes protocol violations in both the study groups). 

Both the treatment groups were well matched with respect to demography and baseline disease characteristics except for the proportion of patients having rhinorrhea and nasal congestion that was more in the IMMBO group. The pediatric population was also well distributed in both treatment groups (Table 1). 

**Table 1:** Baseline measures *Represent values in mean ± SD and two-sample t-test used for the comparison; # represent values in n (%) and Chi-square test used for the comparison; ARIA: Allergic Rhinitis and its Impact on Asthma 2019; IMMBO: Ayurvedic herbo-mineral formulation

	IMMBO group (n = 114)	Montelukast + Levocetirizine group (n = 110)	P value
Age, categorical^#^			
<12 years	10 (8.77%)	7(6.36%)	0.50
≥12 years	104 (91.23%)	103 (93.46%)	
Age, continuous (unit: years) mean ± SD	25.31± 10.21	26.82 ± 9.80	0.26
Gender^#^			
Female	42 (36.84%)	39 (35.45%)	0.83
Male	72 (63.15%)	71 (64.54%)	
Past history of allergic rhinitis (in years), Mean ± SD*	1.00 ± 3.60	1.21 ± 3.97	0.68
ARIA Symptoms^#^			
Sneezing	89 (78.07%)	91 (82.73%)	0.38
Nasal itching	80 (70.18%)	80 (72.73%)	0.67
Nasal congestion	62 (54.39%)	31 (28.18%)	<0.01
Rhinorrhea	50 (43.86%)	12 (10.91%)	<0.01
Conjunctival hyperemia	20 (17.54%)	17 (15.45%)	0.67
Watering of eyes	15 (13.16%)	13 (11.82%)	0.76

At the end of 28 days of therapy, both IMMBO and montelukast + levocetirizine combination showed significant improvement in TNSS score in both all treated population and per protocol population. Reduction in TNSS score was significantly greater in IMMBO-treated patients as compared to the montelukast + levocetirizine combination (Table 2). Complete resolution of nasal symptoms was reported in 92.98% of patients treated with IMMBO vs. 82.73% of patients who received montelukast + levocetirizine combination in all treated patients while nasal symptoms were resolved completely in 90.35% IMMBO treated patients vs. 75.45% patients treated with levocetirizine + montelukast group in per protocol patients. This difference was statistically significant favoring IMMBO in both all treated population and per protocol population (Table 2). 

**Table 2:** Change in total nasal symptom score (TNSS) – all treated patients Data is presented as median and range (min to max); P value* two-sample median test is used;  P value# paired non-parametric test is used; IMMBO: Ayurvedic herbo-mineral formulation

Outcome	IMMBO (n = 114)	Montelukast + Levocetirizine (n = 110)	P value*
TNSS			
Pre	6 (1 to 12)	3 (1 to 10)	< .0001>
Post	0 (0 to 1)	0 (0 to 2)	0.0030
Change	-5 (-12 to 0)	-3 (-10 to -1)	< .0001>
P value#	< .0001>	< .0001>	

Subgroup analysis

Subgroup analysis of TNSS in the pediatric population (age 4 to 12 years) also reported a significant fall in TNSS in both treatment groups. A similar trend was also observed in patients with age more than 12 years (Table 3). 

**Table 3:** Change in total nasal symptom score (TNSS) – agewise stratification Data is presented as median and range (min to max); P value* two-sample median test is used;  P value# paired non-parametric test is used; IMMBO: Ayurvedic herbo-mineral formulation

	Age<12			Age≥12		
Outcome	IMMBO (n =10)	Montelukast + levocetirizine (n =7 )	P value*	IMMBO (n = 104)	Montelukast + levocetirizine (n = 103)	P value*
TNSS						
Pre	3 (2 to 9)	3 (2 to 7)	0.9638	6 (1 to 12)	3 (1 to 10)	< .0001>
Post	0 (0 to 1)	0 (0 to 0)	0.2217	0 (0 to 1)	0 (0 to 2)	0.0009
Change	-3 (-8 to -2)	-3 (-7 to -2)	0.9046	-6 (-12 to 0)	-3 (-10 to -1)	< .0001>
P value#	0.0020	0.0156		< .0001>	< .0001>	

At the end of 28 days of therapy, both IMMBO and montelukast + levocetirizine combination showed significant improvement in IgE levels in both all treated population and per protocol population (Table 4). The reduction in IgE levels favors IMMBO. This reduction in IgE levels was similar irrespective of the age group of the study population (Table 5). 

**Table 4:** Change in Immunoglobin E (IgE) – all treated patients Data is presented as median and range (min to max); P value* two-sample median test is used;  P value# paired non-parametric test is used; IMMBO: Ayurvedic herbo-mineral formulation

Outcome	IMMBO (n = 114)	Montelukast + Levocetirizine (n = 110)	P value*
IgE			
Pre	670 (56 to 3200)	554 (189 to 2158)	0.1600
Post	321 (102 to 1400)	321 (102 to 1400)	1.0000
Change	-226.750 (-2876 to 1244.8)	-195.800 (-1734.8 to 396.2)	0.6410
P value#	<0.0001	<0.0001	

**Table 5:** Change in Immunoglobin E (IgE) – agewise stratification Data is presented as median and range (min to max); P value* two-sample median test is used; P value# paired non-parametric test is used; IMMBO: Ayurvedic herbo-mineral formulation

	Age<12			Age≥12		
Outcome	IMMBO (n =10)	Montelukast + levocetirizine (n = 7)	P value*	IMMBO (n = 104)	Montelukast + levocetirizine (n = 103)	P value*
IgE						
Pre	822 (124 to 1817)	566.2 (321 to 1012)	0.2151	662 (56 to 3200)	551 (189 to 2158)	0.4042
Post	276.6 (222 to 806)	275 (222 to 806)	0.7782	321.9 (102 to 1400)	321 (102 to 1400)	0.9445
Change	-499.950 (-1564.9 to 251))	-206.000 (-415.7 to -99)	0.4990	-205.350 (-2876 to 1244.8)	-194.900 (-1734.8 to 396.2)	0.7810
P value#	0.3438	0.0156		0.0042	< .0001>	

Both the study treatments were well tolerated by the patients. No treatment-emergent adverse events were reported during the study. End of the study laboratory investigation revealed no clinically significant changes. 

## Discussion

Allergic rhinitis is the most common type of rhinitis. It is an inflammation of the nasal membrane that is characterized by sneezing, nasal congestion, nasal itching, and rhinorrhoea in any combination. Peak occurrence of allergic rhinitis is reported in the age group of 20-40 years [18]. In the Indian scenario, approximately 20 to 30 percent population suffers from allergic rhinitis, and out of those 15 percent develop asthma and chronic obstructive pulmonary disease [19]. The highest prevalence of allergic rhinitis is reported in southern regions of India [20]. Allergic rhinitis imposes an economic burden of approximately 3.4 billion in India. Besides the economic impact, it also affects individuals’ social performances, sleep, memory, emotion, and psychology, degrading their quality of life [21].

A combination of levocetirizine and montelukast is effective in treating allergic rhinitis symptoms like congestion, sneezing, itching, and runny nose and provides relief to patients by reducing TNSS and IgE levels. However, they do not generally address recurrences and their prolonged use can cause side effects like drowsiness, dry mouth, fatigue, headaches, gastrointestinal issues, and mood changes [22]. Intranasal corticosteroids also have limitations associated with side effects in bringing relief to allergic rhinitis patients [23]. Newer approaches are needed to treat allergic rhinitis by targeting the underlying immune response for long-term symptom relief with minimal side effects. Current treatments provide relief for some patients but investigating newer therapies that provide a radical cure is important [24].

An Ayurvedic herbo-mineral formulation, IMMBO, was found to be effective in treating patients of allergic rhinitis in a North India-based clinic. Later, the efficacy of IMMBO in patients with allergic rhinitis was reported in non-randomized observational clinical studies conducted by dispensing Ayurvedic physicians across India. The present study was designed to assess its efficacy in a randomized controlled clinical set-up. The results of the study interestingly showed that the 28-day-long treatment using IMMBO brought outcomes comparable to the combination of levocetirizine and montelukast by reducing IgE levels and TNSS score, without any grade II toxicity or reported adverse effects.

IMMBO is developed using *Mandoor Bhasma* with eighteen herbs, following the principles of Ayurveda. There is no information available on its mode of action as yet. This study is the first of its type where traditional Ayurvedic formulation was evaluated in comparison with well-established conventional treatment of allergic rhinitis. The study protocol was executed under the guidance of a subject expert at a premier medical institute in India and the diagnosis of allergic rhinitis was done based on internationally recognized ARIA criteria. The results of the study are encouraging. While both treatments brought significant improvement, IMMBO was significantly better in reducing TNSS and IgE levels (p<0.05). Subgroup analysis and subjects aged below 12 years also revealed that there was a significant fall in TNSS and IgE levels from baseline. This proves that IMMBO is also effective in the pediatric population. There were no serious side effects reported during the study and both treatments were well tolerated. 

The result of the present study established prima facie evidence of the clinical efficacy of IMMBO in treating patients with allergic rhinitis. The result is significant in all aspects. IMMBO could be considered a meritorious formulation for scientific exploration in the treatment of allergic rhinitis.

## Conclusions

This study is unique as it compares the efficacy and tolerability of an Ayurvedic herbo-mineral formulation, IMMBO to well-established allopathic medications for treating allergic rhinitis. The results indicate that the herbo-mineral formulation has the potential to treat allergic rhinitis. IMMBO has promising qualities that warrant further scientific explorations. 
